# Monthly Summary

**Published:** 1889-10

**Authors:** 


### Monthly Summary.

---

A REAL ELIXIR OF YOUTH.—A physician writes to *The Lancet* as follows: " When the season began in May last, a veteran aged sixty, I thought that my tennis days were nearly numbered. A walk of four or five miles tired me. Aching and burning in my great-toe joints made walking somewhat painful. I had had rheumatism in my right shoulder for months, so that I was kept awake at night; acute pain at the insertion of the right deltoid accompanied elevation of the arm, and the left shoulder was also uncomfortable. My first day's practice at tennis was disheartening. I could scarcely serve over the net (underhand). My strokes were extremely feeble, and an overhead stroke caused agony at the insertion of the deltoid. Nevertheless I persevered, and by the end of May, after several good warmings at tennis, I lost all my pains, my joints were freer, I could run about with less aching of the feet, my strokes were more severe, and I could elevate my arm for an overhead stroke without pain. After another month's practice, playing two or three times only in the week, so as not to tire the arm too much, I was fit for tournament play, and I ended the seas n with winning, early in September, an open veterans' singles event. The pleasant exercise and exertion of tennis, taken in moderation, not only drove away stiffness, my threatened rheumatism, and improved my muscular system so that I could walk fifteen miles a day, or play six or eight sets of tennis in an afternoon without undue fatigue, but it cleared my mental faculties, strengthened my perceptions, gave me a better "grasp of the situation," and rendered me more alert in my ordinary work. I still look forward to the enjoyment of future seasons and coming tournaments. A game that will thus renovate a veteran is invaluable from a medical point of view. If used with discretion, it is to the busy man more available and of greater advantage to keep him in condition than cricket, football, or any other game I know, and the accidents that happen to players of lawn tennis are very few, and not of a serious character."

---

SYPHILITIC TEETH.—Referring to syphilitic teeth, Mr. Hutchinson had nothing new to say, but thought that a review of the present state of opinions on the subject might be profitable. He believed that it might now be said that the value of the notches on the upper incisors is fully recognized all over the world as indications of inherited syphilis, and as time had gone on he thought that they had learned to concentrate their attention on the upper central incisors as being the test teeth; other teeth were often more decidedly marked but were less reliable. Respecting the test teeth, an experience of thirty years enabled him to say that, where their malformation had been well characterized he did not think that in a single case he had been misled. With reference to numerous cases of inherited taint in which in spite of it the characteristic teeth—using the word characteristic it its strongest sense—were not present, the best opportunities for observing the range within which syphilite malformations of the teeth might vary and occur, were those in which several members of the same family had suffered from inherited taint. The experience of recent years had confirmed, without in any way explaining, two items in clinical observation which he had made long ago.

The first was, that those subjects of inherited taint who presented good examples of *interstitial keratitis* have almost invariably malformed teeth, and those who have malformed teeth scarcely ever escape *interstitial keratitis.* The second was, that those who are liable to suffer in after-life from phagedenic affections of the mouth or throat, very often, perhaps usually, show nothing peculiar in their teeth.—*Odontographic Journal.*

---

NITROUS OXIDE NARCOSIS AND THE BLOOD.—Dr. A. Rothman writing in the *Vierteljahrsschrift fur Zahnheilkunde,* criticises the communication on the above subject of Dr. Ulbrieh, a translation of which we published in our last issue. He agrees that nitrous oxide cannot form a stable chemical combination with the hæmoglobin as does nitric oxide or carbonic oxide, for were this the case, the blood would not be able to absorb oxygen, and death would rapidly ensue. With reference to the spectral analysis of blood impregnated with nitrous oxide, he quotes Preyer, Buxton, and MacMunn, who asserts that it is precisely the same as that of oxy-hæmoglobin, that is, there are two absorption stiipes marked on the scale between D and E. Making numerous experiments on the human subject, Dr. Rothman found that he was unable to distinguish between the two spectra, but made this observation, that the blood of narcotized persons was more difficult to deduce than ordinary blood, the broad band between D. and E, characteristic of reduced hæmoglobin being longer in appearing after adding sulphate of ammonia in the case of nitrous oxide narcosis blood than when normal blood was used. The broader stripes and the dark aspect of the violet end of the spectrum, as shown in Dr. Ulbrieh's plate, as characteristic of nitrous oxide hæmoglobin, Dr. Rothman believes to be simply a matter of the amount of dilution of the blood examined. The absorption bands of certain pigments will make their appearance in solutions of every possible variety of strength, and always at the same portion of the spectrum, but the breadth, their clearness, and the commencement of their shaded border depend only upon the concentration of the solution and the intensity of the light, Using a Bunsen-Kirchof spectroscope, which allows of the simultaneous examination of two spectra, he found that with one in a thirty-two solution of natural blood and a similiar solution of blood drawn from a person narcotised to the full extent with nitrous oxide, that the spectra very nearly corresponded, and that with further dilution, the breadth of the bands decreased equally in both. He therefore concludes that in the inhalation of nitrous oxide there is no chemical combination with the hæmoglobin.—*Dental Record.*

---

AXIOMS IN PROSTHETIC DENTISTSY.—*By Dr. L. P. Haskell.*—1. If many teeth have been lost, and the patient has reached that condition when an artificial denture must be worn, the first thing to be considered is: "What must be done to make the denture as *useful* and *comfortable* as possible?" If the extraction of other teeth will secure this result, extract them.

2. When artificial teeth are required, there is nothing in which skill is so important to personal appearance, health and comfort. Remind your patient of this when she is "shopping" for them.

3. The more difficult it may be to secure an impression, the greater the necessity for the use of plaster.

4. There are more failures from faulty articulation than from misfits.

5. Never allow the front teeth to meet, except in the rare cases where the protrusion of the lower jaw brings the upper teeth *inside.*

6. Never allow a lower second or third molar, which has been thrown foward, so its crown stands at an angle of about 45°, to be met on its face by an upper tooth.

7. When the cuspids have been extracted a year, there is always room and necessity for the plate to be worn higher and the gum fuller, to restore the contour of the lip.

8. There is no necessity for vacuum cavities in full upper dentures, whatever the shape or condition of the alveolar ridge or palate.

9. The nearer the karat of the solder to that of the plate the more satisfactory will be the results. No lower karat than 18 need be, nor should be, used.

10. A full set of single gum teeth, *soldered* to a gold plate, in these days of rubber attachments and continuous gum work is an abomination from every point of view, and the teaching of it to a student is time wasted, foolishly wasted.

11. "Combination" dentures of any kind, which cannot be repaired without making almost a new denture (and there are such), are an imposition on the patient.—*Items of Interest.*

---

COCAINE HALLUCINATIONS.—MM. Magnan and Saury report three cases of hallucination due to the cocaine habit. One patient was always scraping his tongue, and thought he was extracting from it little black worms; another made his skin raw in the endeavor to draw out cholera microbes; and a third, a physician, is perpetually looking for cocaine crystals under his skin. Two patients suffered from epileptic attacks, and a third from cramps. It is important to notice that two of these patients were persons who had resorted to cocaine in the hope of being able to cure themselves thereby of the morphine habit, an expectation which had been disappointed. For more than a year they had daily injected from one to two grammes of cocaine under the skin, without, however, giving up the morphine injections, which were only reduced in quantity. The possibility of substituting cocainism in the endeavor to cure morphinomania is a danger, therefore, which must be carefully held in view.—*British Medical Journal.*

---

DEATH AFTER TAKING NITROUS OXIDE.—A painful death occurred in a dentist's house in Edinburgh recently. An old lady, of over 75, visited a dentist for the purpose of having a tooth removed for disease of the antrum. The operation was successfully conducted with the aid of nitrous oxide, but, while the cavity was being treated, the patient was observed to lapse into a state of unconsciousness. All efforts at resuscitation proved ineffectual and, before further medical advice could be obtained, death had resulted. It appears that the patient had suffered previously from weak cardiac action.—"British Medical Journal," Oct. 12, 1889.

